# Algal Cultivation for Obtaining High-Value Products, 2nd Edition

**DOI:** 10.3390/md24090304

**Published:** 2026-08-31

**Authors:** Cecilia Faraloni, Eleftherios Touloupakis

**Affiliations:** 1 Istituto per la Bioeconomia, Consiglio Nazionale delle Ricerche, Via Madonna del Piano 10, Sesto Fiorentino, 50019 Firenze, Italy; 2 Istituto di Ricerca sugli Ecosistemi Terrestri, Consiglio Nazionale delle Ricerche, Via Madonna del Piano 10, Sesto Fiorentino, 50019 Firenze, Italy

The growing demand for natural products in the pharmaceutical, nutraceutical, cosmetic, food, and feed sectors has stimulated considerable interest in the identification and sustainable production of bioactive compounds from renewable biological resources. Increasing consumer awareness of environmental sustainability and the potential adverse effects of synthetic additives has further accelerated the search for naturally derived ingredients with functional and health-promoting properties. In this context, photosynthetic microorganisms such as microalgae and cyanobacteria have emerged as some of the most promising sources of high-value compounds because of their extraordinary biochemical diversity, rapid growth rates, and ability to thrive under a wide range of environmental conditions.

Microalgae inhabit diverse ecosystems and are frequently exposed to environmental stresses such as high irradiance, salinity fluctuations, nutrient limitation, temperature extremes, and ultraviolet radiation. Through evolution, these photosynthetic microorganisms have developed sophisticated adaptive mechanisms that enable survival under such challenging conditions. Many of these adaptations involve the synthesis and accumulation of secondary metabolites that protect cells from oxidative damage and environmental stress. As a result, microalgae represent a rich reservoir of biologically active molecules, including carotenoids, polyphenols, pigments, polysaccharides, vitamins, fatty acids, and other antioxidant compounds. Among the various classes of microalgal metabolites, antioxidant compounds have received particular attention because of their demonstrated ability to neutralize reactive oxygen species and reduce oxidative stress, which is associated with numerous chronic diseases and aging-related processes. Moreover, many microalgal-derived compounds exhibit additional biological activities, including anti-inflammatory, antimicrobial, antiviral, anticancer, and immunomodulatory properties. These multifunctional characteristics make microalgae attractive candidates for the development of innovative products aimed at improving health while simultaneously supporting the transition toward more sustainable industrial practices.

The production of high-value metabolites by microalgae is strongly influenced by the strain used, genetic diversity, and cultivation conditions. Environmental factors such as light intensity, temperature, and nutrient availability can significantly affect metabolic pathways, often stimulating the accumulation of compounds with antioxidant and protective functions. Consequently, research in recent years has increasingly focused on understanding the complex interactions between environmental stress and metabolite biosynthesis, as well as on developing cultivation strategies that enhance productivity and product quality. Advances in biotechnology, process engineering, and systems biology have further enabled more efficient exploitation of microalgal resources, opening new opportunities for large-scale production and commercialization. However, further research is needed to improve strain selection and genetic characterization, optimize cultivation systems under variable environmental conditions, and develop cost-effective extraction technologies that preserve bioactive compound quality. Addressing these challenges will be crucial for translating scientific discoveries into commercially viable solutions that meet growing market demands.

The objective of this Special Issue was to highlight recent advances in the discovery, production, extraction, characterization, and application of high-value compounds derived from microalgae. This section offers a concise summary of each of the ten contributions to this Special Issue. The contributions are listed in the List of Contributions.

Contributor 1 investigated seven microalgal species and an isolated mixed culture to evaluate their potential as biostimulants and biofertilizers under autotrophic cultivation conditions and to identify the most promising species as an alternative to synthetic fertilizers.

In contribution 2, Dobrinčić et al. examined the effects of various carbon and nitrogen sources on the growth and lipid metabolism of *Desmodesmus subspicatus*, focusing on ryegrass enzymatic hydrolysates as an alternative carbon source. Their results support ryegrass as a viable alternative carbon source and highlight cultivation parameters that influence growth and lipid quality relevant to biofuel applications.

In contribution 3, Alemán et al. evaluated and optimized the cultivation of *Limnospira platensis* BEA 1257B in seawater, demonstrating the feasibility of long-term cultivation with medium recirculation under full seawater conditions. Their results highlight its potential for sustainable marine biotechnology, supporting food production in coastal areas with limited agricultural resources and freshwater availability, and contributing to improved food security.

Contributor 4 studied the effects of different light intensities and salt concentrations on the growth and biomass composition of *Synechocystis.* The results showed that *Synechocystis* could grow at all light intensities and marine salt concentrations through increased carotenoid synthesis in response to physiological stress.

Contributor 5 investigated how salinity and pH modulation affect the growth, biochemical composition, and bioactive compound production of *Limnospira platensis* under photoautotrophic batch cultivation. The findings highlight the potential of *Limnospira platensis* as a valuable source of proteins, pigments, and antioxidants, and emphasize the utility of moderate environmental stress for enhancing biomass quality.

In contribution 6, Yang et al. used *Phaeodactylum tricornutum* to identify optimal conditions for fucoxanthin accumulation through a three-factor, four-level orthogonal design. Their results showed that glycine, light intensity, and photoperiod synergistically regulate the expression of genes involved in photosynthesis and carotenoid biosynthesis, thereby enhancing fucoxanthin accumulation and providing a theoretical basis for optimizing fucoxanthin production.

Contributor 7 investigated the effects of titanium dioxide, zinc oxide, and copper oxide nanoparticles on biomass growth, astaxanthin biosynthesis, and lipid accumulation in *Haematococcus lacustris*, with particular emphasis on their stage-specific impact throughout the algal life cycle. The results showed that these nanoparticles modulate growth and astaxanthin biosynthesis in a life cycle-dependent manner.

Contributor 8 investigated the potential of *Phaeodactylum tricornutum* as a sustainable, nutritious food source, highlighting its ability to produce valuable bioactive compounds, including eicosapentaenoic acid, fucoxanthin, chrysolaminarin, and proteins. The study also established a foundation for future photobioreactor optimization to improve production efficiency and tailor biomass composition for specific health-promoting applications.

In contribution 9, the authors overview the convergence of CRISPR-Cas technologies in microalgae research, highlighting their impact on genetic studies, metabolic engineering, and industrial applications. They summarize recent advances in microalgal genome editing through CRISPR systems, outline current technical challenges, and discuss future directions for improving the implementation of this innovative technology in microalgal biotechnology.

In contribution 10, the authors review CO_2_ supplementation technologies used in photobioreactor systems, focusing on different mechanisms for enhancing CO_2_ mass transfer and evaluating the effectiveness of these technologies while exploring their potential for scale-up. Among these strategies, membrane-based CO_2_ delivery systems and the use of CO_2_ absorption enhancers have shown the highest efficiency in improving CO_2_ mass transfer and microalgae productivity.

In conclusion, the contributions in this Special Issue provide valuable insights into the potential of microalgae as biofactories of high-value compounds. Together, they highlight the scientific advances and technological innovations that are driving the field forward. This collection aims to encourage further research, promote interdisciplinary collaborations, and advance the creation of innovative microalgal-based products that enhance human health, industrial sustainability, and the burgeoning bioeconomy.

We extend our heartfelt appreciation to all authors for their significant contributions, to the reviewers for their expertise and commitment to maintaining the quality of the published work, and to the editorial staff for their professional assistance in preparing this Special Issue.

